# The Prognostic Value of Preoperative Serum Tumor Markers in Non-Small Cell Lung Cancer Varies With Radiological Features and Histological Types

**DOI:** 10.3389/fonc.2021.645159

**Published:** 2021-06-11

**Authors:** Haiqing Chen, Fangqiu Fu, Yue Zhao, Haoxuan Wu, Hong Hu, Yihua Sun, Yawei Zhang, Jiaqing Xiang, Yang Zhang

**Affiliations:** ^1^ Department of Thoracic Surgery and State Key Laboratory of Genetic Engineering, Fudan University Shanghai Cancer Center, Shanghai, China; ^2^ Institute of Thoracic Oncology, Fudan University, Shanghai, China; ^3^ Department of Oncology, Shanghai Medical College, Fudan University, Shanghai, China

**Keywords:** non-small cell lung cancer, serum tumor marker, prognosis, ground glass opacity, recurrence free survival

## Abstract

**Objectives:**

To assess the association between common-used serum tumor markers and recurrence of lung adenocarcinoma and squamous cell carcinoma separately and determine the prognostic value of serum tumor markers in lung adenocarcinoma featured as ground glass opacities.

**Methods:**

A total of 2,654 non-small cell lung cancer patients undergoing surgical resection between January 2008 and September 2014 were analyzed. The serum levels of carcinoma embryonic antigen (CEA), cytokeratin 19 fragment (CYFRA21-1), neuron-specific enolase (NSE), carbohydrate antigen 125 (CA125), carbohydrate antigen 153 (CA153) and carbohydrate antigen 199 (CA199) were tested preoperatively. Survival analyses were performed with COX proportional hazard regression.

**Results:**

Among patients with lung adenocarcinoma, elevated preoperative serum CEA(HR=1.246, 95%CI:1.043-1.488, P=0.015), CYFRA21-1(HR=1.209, 95%CI:1.015-1.441, P=0.034) and CA125(HR=1.361, 95%CI:1.053-1.757, P=0.018) were significantly associated with poorer recurrence free survival (RFS). Elevated preoperative serum CA199 predicted worse RFS in patients diagnosed with lung squamous cell carcinoma (HR=1.833, 95%CI: 1.216-2.762, P=0.004). Preoperative serum CYFRA21-1(HR=1.256, 95%CI:1.044-1.512, P=0.016) and CA125(HR=1.373, 95%CI: 1.050-1.795, P=0.020) were independent prognostic factors for patients with adenocarcinoma presenting as solid nodules while serum CEA (HR=2.160,95%CI:1.311-3.558, P=0.003) and CA125(HR=2.475,95%CI:1.163-5.266, P=0.019) were independent prognostic factors for patients with adenocarcinoma featured as ground glass opacities.

**Conclusions:**

The prognostic significances of preoperative serum tumor markers in non-small cell lung cancer were associated with radiological features and histological types.

## Introduction

Various serum tumor markers had been reported in the diagnosis and prognosis prediction of miscellaneous cancers. Despite the inefficiency of serum tumor markers in the early diagnosis of non-small cell lung cancer (NSCLC), the prognostic significances of several serum tumor markers had been reported in a series of studies (1–6). But it still remained controversial as the other studies reported contrary results (7–9). Few studies had evaluated the prognosis of multiple serum tumor markers in a single research systemically, the discrepancy in the serum tumor makers included might lead to the conflicting results. Although lung adenocarcinoma (ADC) and squamous cell carcinoma (SCC) were distinct in the clinical and genomic characteristics (10–14), only several studies analyzed ADC and SCC respectively (15, 16) and the other studies always analyzed them together under the category of NSCLC. The difference in the inherent production mechanism and function of different serum tumor markers indicated that they might play different roles in the prognosis prediction of lung ADC and lung SCC. The conflicting results might also be caused by the different ratios of ADC/SCC across these studies. The prognosis significances of serum tumor markers in lung adenocarcinoma and lung SCC still required large-sample study to clarify.

With the increasing application and decreasing cost of low-dose computed tomography (CT), more and more lung nodules characterized by ground glass opacity (GGO) were detected (17). In general, patients with lung cancer manifesting as GGO showed a good prognosis (18, 19). Previously, we have demonstrated that lung adenocarcinoma manifesting as GGO defined a special clinical subtype (20). We also found distinct prognostic factors in patients with GGO-featured and radiologically solid adenocarcinoma (21). But no study had investigated the correlation between evaluated serum tumor markers and prognosis in lung adenocarcinoma presenting as GGO.

Hence, this study aimed to systemically investigated the prognostic significances of six common-used serum tumor makers in surgically treated lung adenocarcinoma and SCC respectively and explore the prognostic significances of serum tumor markers in lung adenocarcinoma presenting as GGO.

## Methods

**Figure 1:**
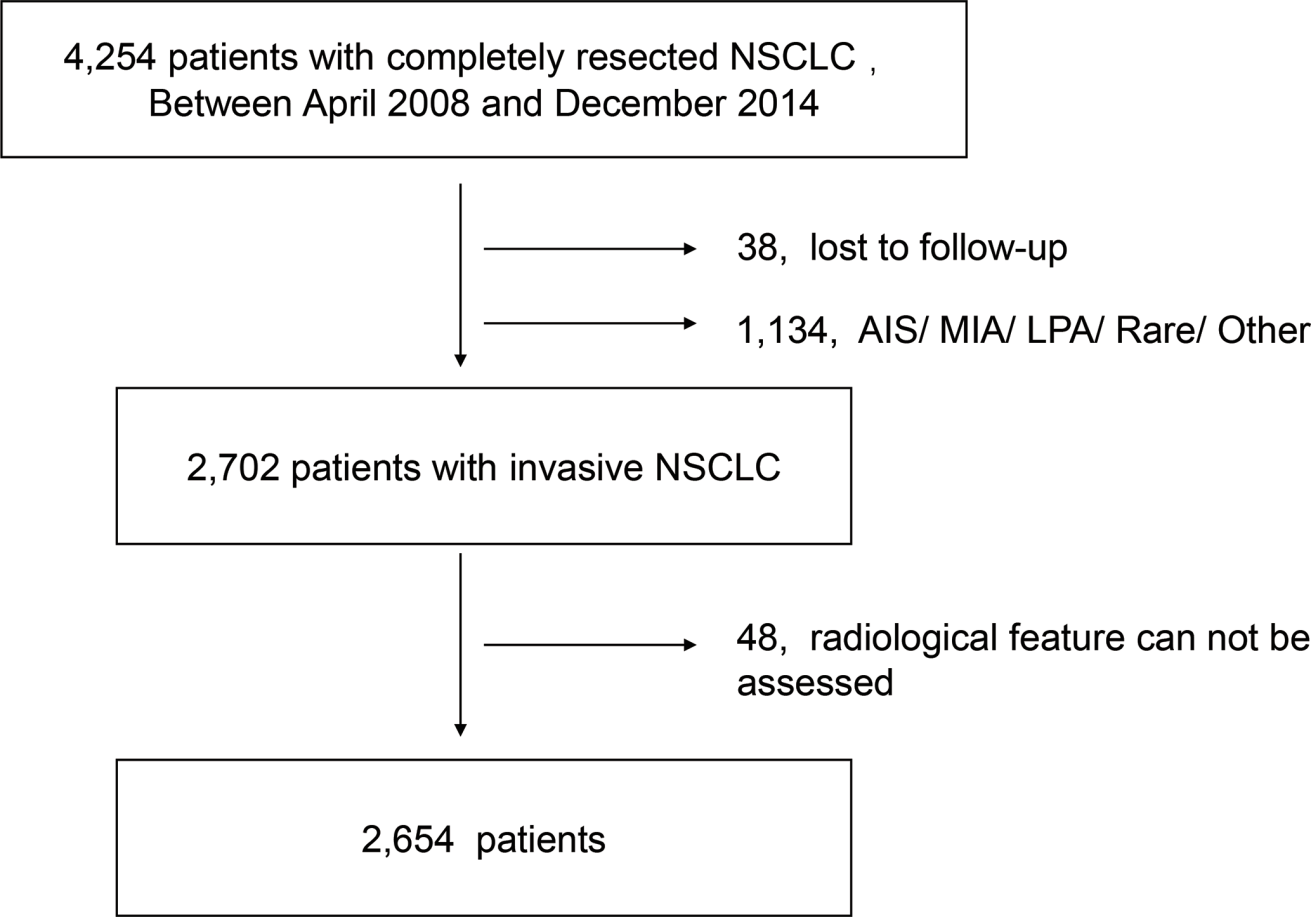
Flow diagram of patients inclusion and exclusion.

We retrospectively reviewed the data of NSCLC patients without a history of previous malignant tumor who underwent pulmonary surgery at Fudan University Shanghai Cancer Center (FUSCC) between January 2008 and September 2014. The exclusion criteria including missing information of follow-up, incomplete data of serum tumor markers (CEA, CYFRA21-1, NSE, CA199, CA153, CA125) and missing radiological feature information. Since no recurrence was detected in patients diagnosed with adenocarcinoma *in situ*, minimal invasive adenocarcinoma and lepidic predominant adenocarcinoma, these patients therefore were excluded. Also, patients diagnosed with enteric adenocarcinoma, fetal adenocarcinoma and NSCLC other than ADC and SCC were excluded because their small numbers and varied histological types (**Figure 1**). According to the previous studies, acinar predominant adenocarcinoma, papillary predominant adenocarcinoma, and invasive mucinous adenocarcinoma were categorized as low-grade adenocarcinoma (LGADC), while micropapillary predominant adenocarcinoma and solid predominant adenocarcinoma were categorized as high-grade adenocarcinoma (HGADC) (22–24). In our institution, GGO component was defined as an area of a slight, homogenous increase in density that did not obscure the underlying vascular markings (25). The maximum diameter on the single largest axial dimension was measured on a lung window, and an edge-enhancing (sharp) filter was recorded for the size of solid component and whole nodule. Consolidation to tumor ratio (CTR) was defined as the ratio of the maximum size of solid component to the maximum tumor size on the thin-section CT scan in the axial plane (26).

Patients were followed up with chest CT, ultrasonography of abdominal and supraclavicular region and brain magnetic resonance imaging every 4 months for the first 3 years, every 6 months for the 3 to 5 years and then annually. Bone scintigraphy was performed annually. The examinations were also performed when related symptoms occurred. The median follow-up time of the whole patient cohort was 41 months which might lead to inadequate overall survival events for analysis especially for GGO-featured adenocarcinoma. Hence, we focused on the recurrence free survival of these patients. Recurrence free survival was defined as the time interval between the date of surgery and recurrence or the last follow-up.

Statistics analyses were conducted using SPSS 22.0 for Windows (Chicago, USA). and R 3.50 (Vienna, Austria). The upper limit values of serum tumor markers concentrations were defined according to the manufacturers. They were determined based on the 95% specificity level for normal population who received serum test during the reference ranges determination procedure by the central laboratory of FUSCC (5.20ng/ml for CEA, 16.30ng/ml for NSE, 2.66ng/ml for CYFRA21-1, 35.00U/ml for CA125, 25.00U/ml for CA153 and 27.00U/ml for CA199). Patients were divided into two groups (Elevated/Normal) according to the upper limit values respectively. The association of categorized variables were tested using Pearson’s Chi-Square test. The differences of survival between the two groups were presented with Kaplan-Meier curves and tested with log-rank statistics. The demographics of patients (sex, age, smoking history), radiological features (consolidation to tumor ratio), treatment details (surgical procedures), characteristics of tumors (lymph vascular invasion, visceral pleural invasion, pathological size, histological subclassification, T classification, N classification) and the levels of preoperative serum tumor markers (CEA, CYFRA21-1, NSE, CA125, CA153 and CA199) were included in the survival analyses. Variables with a P value <0.1 in the univariable analyses were included in the multivariable analyses. Multivariable COX proportional hazard regression analyses with backward stepwise elimination were performed to identify the independent prognostic factors.

The study was approved by the Institutional review board (IRB) of FUSCC. Since the study was retrospective and the data were anonymous, the informed consent was waived by the IRB.

## Results

The data of a total of 4,254 patients were reviewed. According to the exclusive criteria, 2,654 patients were included in the final analyses including 1,071 female patients and 1,583 male patients. The median age of this cohort was 61 (interquartiles, 55-67). 73.5% patients were diagnosed with radiologically solid tumors and 26.5% patients with ground glass opacities on the radiograph. The majority of the patients (96.8%) received lobar resection. 27.9% patients were diagnosed with SCC. 12.3% patients were diagnosed with HGADC and 59.8% patients were diagnosed with LGADC. More detailed characteristics of the cohorts were shown in the **Table 1**.

**Table 1:** Clinicopathological characteristics of the patient cohorts.

Variables		NSCLC (2,654)	ADC (1,914)	SCC (740)
Sex, n (%)	Female	1071 (40.4%)	1028 (53.7%)	43 (5.8%)
	Male	1583 (59.6%)	886 (46.3%)	697 (94.2%)
Age (yr.)	median (quartiles)	61 (55-67)	61 (54-67)	62 (56-68)
Smoking History, n (%)	Never	1447 (54.5%)	1299 (67.9%)	148 (20.0%)
	Yes	1207 (45.5%)	615 (32.1%)	592 (80.0%)
Radiological feature, n (%)	pGGO	71 (2.7%)	71 (3.7%)	0
	mGGO	633 (23.9%)	598 (31.2%)	35 (4.7%)
	Solid	1950 (73.5%)	1245 (65.0%)	705 (95.3%)
Surgery, n (%)	Sublobar	86 (3.2%)	83 (4.3%)	3 (0.4%)
	Lobar	2568 (96.8%)	1831 (95.7%)	737 (99.6%)
LVI, n (%)	Absent	2263 (85.3%)	1624 (84.8%)	639 (86.4%)
	Present	391 (14.7%)	290 (15.2%)	101 (13.6%)
VPI, n (%)	Absent	2095 (78.9%)	1457 (76.1%)	638 (86.2%)
	Present	559 (21.1%)	457 (23.9%)	102 (13.8%)
p-Size (cm)	median (quartiles)	2.5 (1.8-4.0)	2.3 (1.6-3.0)	4.0 (3.0-5.5)
Histology, n (%)	SCC	740 (27.9%)	0	740 (100%)
	HGADC	327 (12.3%)	327 (17.1%)	0
	LGADC	1587 (59.8%)	1587 (82.9%)	0
T, n (%)	T1	1288 (48.5%)	1201 (62.7%)	185 (25.0%)
	T2	1044 (39.3%)	610 (31.9%)	427 (57.7%)
	T3	217 (8.2%)	87 (4.5%)	114 (15.4%)
	T4	105 (4.0%)	16 (0.8%)	14 (1.9%)
N, n (%)	N0	1755 (66.1%)	1307 (68.3%)	448 (60.5%)
	N1	336 (12.7%)	189 (9.9%)	147 (19.9%)
	N2	563 (21.2%)	418 (21.8%)	145 (19.6%)
CEA, n (%)	Elevated	694 (26.1%)	568 (29.7%)	126 (17.0%)
	Normal	1960 (73.9%)	1346 (70.3%)	614 (83.0%)
CYFRA21-1, n (%)	Elevated	1234 (46.5%)	666 (34.8%)	568 (76.8%)
	Normal	1420 (53.5%)	1248 (65.2%)	172 (23.2%)
NSE, n (%)	Elevated	286 (10.8%)	154 (8.0%)	132 (17.8%)
	Normal	2368 (89.2%)	1760 (92.0%)	608 (82.2%)
CA125, n (%)	Elevated	229 (8.6%)	151 (7.9%)	78 (10.5%)
	Normal	2425 (91.4%)	1763 (92.1%)	662 (89.5%)
CA153, n (%)	Elevated	191 (7.2%)	141 (7.4%)	50 (6.8%)
	Normal	2463 (92.8%)	1773 (92.6%)	690 (93.2%)
CA199, n (%)	Elevated	269 (10.1%)	197 (10.3%)	71 (9.7%)
	Normal	2385 (89.9%)	1717 (89.7%)	668 (90.3%)

NSCLC, non-small cell lung cancer; ADC, adenocarcinoma; SCC, squamous cell carcinoma; pGGO, pure ground glass opacity; mGGO, mixed ground glass opacity; LGADC, low grade adenocarcinoma; HGADC, high grade adenocarcinoma.

### Correlation Between Serum Tumor Markers and Clinicopathological Characteristics

**Table 2** listed the correlation between the levels of preoperative serum tumor markers and clinicopathological characteristics. Higher proportions of patients with elevated CYFRA21-1 (P<0.001) and CA125 (P<0.001) were observed in male patients while more female patients were detected with elevated NSE (P<0.001). All the six serum tumor markers were associated with T classifications, N classifications and histological types except that no significant difference of serum CA199 was observed in patients with different histological types (P=0.067).

**Table 2:** Correlation between serum tumor markers and clinicopathological parameters of patients with non-small cell lung cancer.

Variables		CEA			CYFRA21-1			NSE			CA125			CA153			CA199		
		Elevated	Normal	P	Elevated	Normal	P	Elevated	Normal	P	Elevated	Normal	P	Elevated	Normal	P	Elevated	Normal	P
Sex	Female	271(25.3%)	800(74.7%)	0.415	336(31.4%)	735(68.6%)	<0.001	79(7.4%)	992(92.6%)	<0.001	64(6.0%)	1007(94.0%)	<0.001	75(7.0%)	996(93.0%)	0.751	118(11.0%)	953(89.0%)	0.216
	Male	423(26.7%)	1160(73.3%)		898(56.7%)	685(43.3%)		207(13.1%)	1376(86.9%)		165(10.4%)	1418(89.6%)		116(7.3%)	1467(92.7%)		151(9.5%)	1432(90.5%)	
T				<0.001			<0.001			<0.001			<0.001			<0.001			0.005
	T1	292(21.1%)	1094(78.9%)		455(32.8%)	931(67.2%)		80(5.8%)	1306(94.2%)		57(4.1%)	1329(95.9%)		53(3.8%)	1333(96.2%)		118(8.5%)	1268(91.5%)	
	T2	317(30.6%)	720(69.4%)		595(57.4%)	442(42.6%)		125(12.1%)	912(87.9%)		110(10.6%)	927(89.4%)		100(9.6%)	937(90.4%)		116(11.2%)	921(88.8%)	
	T3	77(38.35)	124(61.7%)		161(80.1%)	40(19.9%)		71(35.3%)	130(64.7%)		58(28.9%)	143(71.1%)		32(15.9%)	169(84.1%)		32(15.9%)	169(84.1%)	
	T4	8(26.7%)	22(73.3%)		23(76.7%)	7(23.3%)		10(33.3%)	20(66.7%)		4(13.3%)	26(86.7%)		6(20.0%)	24(80.0%)		3(10.0%)	27(90.0%)	
N				<0.001			<0.001			<0.001			<0.001			<0.001			<0.001
	N0	320(18.2%)	1435(81.8%)		735(41.9%)	1020(58.1%)		157(8.9%)	1598(91.1%)		96(5.5%)	1659(94.5%)		85(4.8%)	1670(95.2%)		151(8.6%)	1604(91.4%)	
	N1	111(33.0%)	225(67.0%)		191(56.8%)	145(43.2%)		53(15.8%)	283(84.2%)		39(11.6%)	297(88.4%)		23(6.8%)	313(93.2%)		35(10.4%)	301(89.6%)	
	N2	263(46.7%)	300(53.3%)		308(54.7%)	255(45.3%)		76(13.5%)	487(86.5%)		94(16.7%)	469(83.3%)		83(14.7%)	480(85.3%)		83(14.7%)	480(85.3%)	
Histologic type				<0.001			<0.001			<0.001			<0.001			<0.001			0.067
	HGADC	129(39.4%)	198(60.6%)		152(46.5%)	175(53.5%)		53(16.2%)	274(83.8%)		53(16.2%)	274(83.8%)		41(12.5%)	286(87.5%)		45(13.8%)	282(86.2%)	
	LGADC	439(27.7%)	1148(72.3%)		514(32.4%)	1073(67.6%)		101(6.4%)	1486(93.6%)		98(6.2%)	1489(93.8%)		100(6.3%)	1487(93.7%)		152(9.6%)	1435(90.4%)	
	SCC	126(17.0%)	614(83.0%)		568(76.8%)	172(23.2%)		132(17.8%)	608(82.2%)		78(10.5%)	662(89.5%)		50(6.8%)	690(93.2%)		72(9.7%)	668(90.3%)	

HGADC, high grade adenocarcinoma; LGADC, low grade adenocarcinoma; SCC, squamous cell carcinoma.

### Recurrence Free Survival of Patients With Elevated Serum Tumor Markers

Recurrence was observed in 799 patients. The correlation of elevated serum tumor markers with recurrence free survival was depicted with Kaplan-Meier curves and tested with log-rank tests.

**Figure 2:**
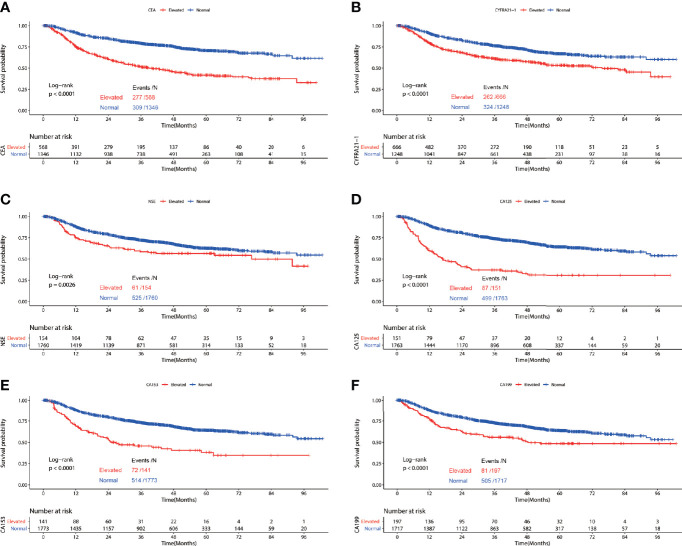
Recurrence free survival of patients with elevated *vs*. normal preoperative serum CEA **(A)**, NSE **(B),** CYFRA21-1 **(C)**, CA125 **(D)**, CA153 **(E)** and CA199 **(F)** in lung adenocarcinoma.

Among patients diagnosed with adenocarcinoma, patients with elevated preoperative serum tumor markers showed significantly worse RFS compared with those with normal serum tumor markers (CEA: P<0.0001, CYFRA21-1: P<0.0001, NSE: P=0.0026, CA153: P<0.0001, CA125: P<0.0001, CA199: P<0.0001) (**Figure 2**). Multivariable COX proportional hazard regression showed that elevated serum CEA (HR=1.246, 95%CI:1.043-1.488, P=0.015), CYFRA21-1(HR=1.209, 95%CI:1.015-1.441, P=0.034) and CA125(HR=1.361, 95%CI:1.053-1.757, P=0.018) were associated with poorer RFS significantly (**Table 3**).

**Table 3:** Association between serum tumor markers and recurrence free survival of patients with lung adenocarcinoma.

		Univariable		Multivariable	
		HR	P	HR	P
Sex	Male *vs*. Female	1.480 (1.258-1.741)	<0.001	1.259 (1.066-1.486)	0.007
Age		0.998 (0.990-1.006)	0.621		
Smoking History	Yes *vs*. Never	1.413 (1.195-1.670)	<0.001		
CTR			<0.001		
	CTR<0.5	1		1	
	0.5<=CTR<1	5.442 (2.504-11.825)	<0.001	4.065 (1.867-8.852)	<0.001
	CTR=1	15.364 (7.286-32.395)	<0.001	5.844 (2.736-12.482)	<0.001
Surgery	Lobar *vs*. Sublobar	2.057 (1.417-2.986)	<0.001		
LVI	Present *vs*. Absent	3.121 (2.598-3.750)	<0.001	1.456 (1.196-1.772)	0.001
VPI	Present *vs*. Absent	1.880 (1.586-2.228)	<0.001	1.333 (1.122-1.583)	<0.001
p-Size		1.330 (1.282-1.379)	<0.001	1.178 (1.119-1.240)	<0.001
Histology	HGADC *vs*. LGADC	1.469 (1.339-1.611)		1.601 (1.220-2.101)	0.001
T			<0.001		
	T1	1			
	T2	2.390 (2.014-2.837)	<0.001		
	T3	4.120 (3.050-5.564)	<0.001		
	T4	6.099 (3.133-11.872)	<0.001		
N			<0.001		<0.001
	N0	1		1	
	N1	2.849 (2.200-3.690)	<0.001	1.601 (1.220-2.101)	0.001
	N2	5.848 (4.905-6.971)	<0.001	3.308 (2.704-4.048)	<0.001
CEA	Elevated *vs*. Normal	1.642 (1.514-1.781)	<0.001	1.246 (1.043-1.488)	0.015
CYFRA21-1	Elevated *vs*. Normal	1.324 (1.221-1.437)	<0.001	1.209 (1.015-1.441)	0.034
NSE	Elevated *vs*. Normal	1.499 (1.150-1.955)	0.003		
CA125	Elevated *vs*. Normal	1.837 (1.638-2.060)	<0.001	1.361 (1.053-1.757)	0.018
CA153	Elevated *vs*. Normal	1.583 (1.399-1.791)	<0.001		
CA199	Elevated *vs*. Normal	1.284 (1.049-1.571)	0.015		

HR, hazard ratio; CTR, consolidation to tumor ratio; LVI, lymph vascular invasion; VPI, visceral pleural invasion; LGADC, low grade adenocarcinoma; HGADC, high grade adenocarcinoma.

**Figure 3:**
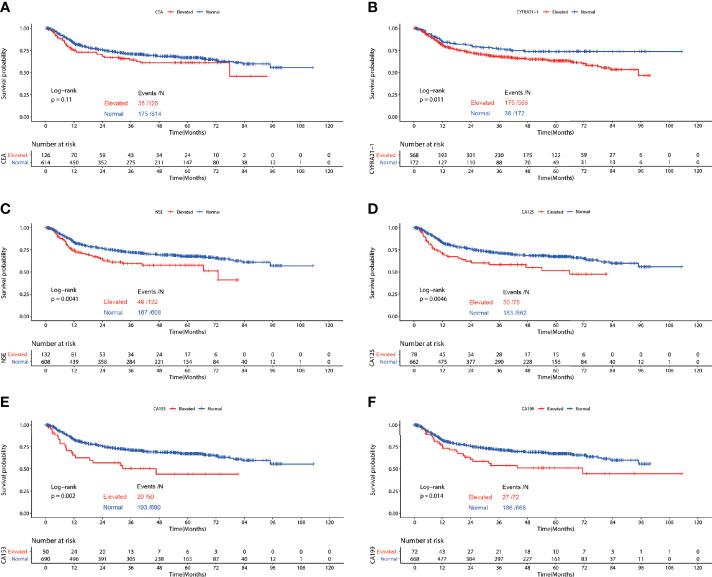
Recurrence free survival of patients with elevated *vs*. normal preoperative serum CEA **(A)**, NSE **(B),** CYFRA21-1 **(C)**, CA125 **(D)**, CA153 **(E)** and CA199 **(F)** in lung squamous cell carcinoma.

In the cohort of patients diagnosed with squamous cell carcinoma, worse RFS was observed in patients with elevated preoperative serum CYFRA21-1(P=0.011), NSE(P=0.0041), CA125(P=0.0046), CA153(P=0.002) and CA199(P=0.014). No significant difference of RFS was observed in patients with elevated CEA and normal CEA(P=0.11) (**Figure 3**). Multivariable COX regression revealed that only elevated preoperative CA199 predicted worse RFS in patients diagnosed with SCC (HR=1.833, 95%CI: 1.216-2.762, P=0.004) (**Table 4**).

**Table 4:** Association between serum tumor markers and recurrence free survival of patients with lung squamous cell carcinoma.

		Univariable		Multivariable	
		HR	P	HR	P
Sex	Male *vs* Female	1.240 (0.676-2.275)	0.488		
Age		1.004 (0.988-1.020)	0.643		
Smoking History	Yes *vs*. Never	0.964 (0.688-1.351)	0.830		
Surgery	Lobar *vs*. Sublobar	1.082 (0.405-2.891)	0.874		
LVI	Present *vs*. Absent	1.809 (1.298-2.523)	<0.001		
VPI	Present *vs*. Absent	2.260 (1.637-2.119)	<0.001	1.790 (1.287-2.491)	0.001
p-Size		1.181 (1.119-1.246)	<0.001	1.140 (1.075-1.208)	<0.001
CTR					
	0.5=<CTR1<1	1			
	1	1.385 (0.615-3.120)	0.432		
T			<0.001		
	T1	1			
	T2	1.609 (1.127-2.298)	0.009		
	T3	2.558 (1.665-3.930)	<0.001		
	T4	2.665 (0.952-7.463)	0.062		
N			<0.001		<0.001
	N0	1		1	
	N1	1.483 (1.020-2.155)	0.039	1.374 (0.943-2.002)	0.098
	N2	3.610 (2.672-4.878)	<0.001	3.015 (2.221-4.092)	<0.001
CEA	Elevated *vs*. Normal	1.152 (0.967-1.374)	0.114		
CYFRA21-1	Elevated *vs*. Normal	1.253 (1.051-1.494)	0.012		
NSE	Elevated *vs*. Normal	1.269 (1.077-1.495)	0.004		
CA125	Elevated *vs*. Normal	1.318 (1.086-1.599)	0.005		
CA153	Elevated *vs*. Normal	1.428 (1.134-1.798)	0.002		
CA199	Elevated *vs*. Normal	1.284 (1.049-1.571)	0.015	1.354 (1.103-1.662)	0.004

HR, hazard ratio; CTR, consolidation to tumor ratio; LVI, lymph vascular invasion; VPI, visceral pleural invasion.

To determine the prognosis value of serum tumor markers in adenocarcinoma presenting as solid nodules and GGO respectively, survival analyses were conducted in the two cohorts separately.

**Figure 4:**
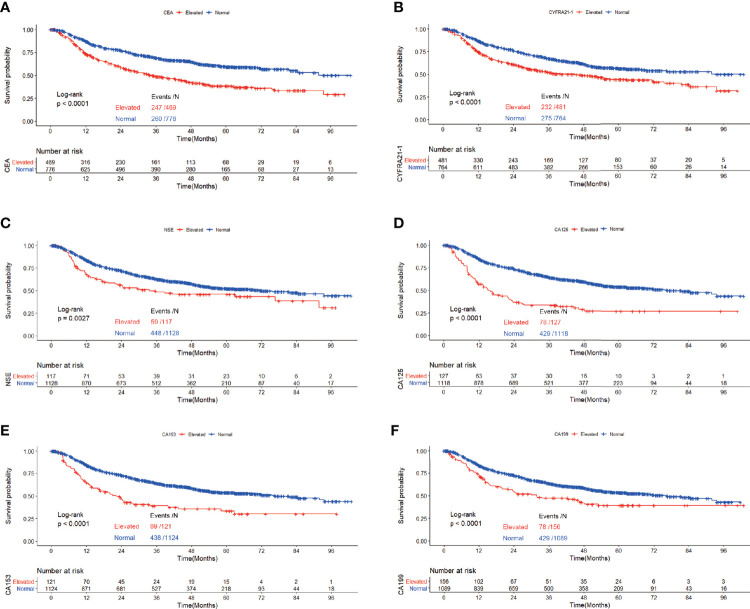
Recurrence free survival of patients with elevated *vs*. normal preoperative serum CEA **(A)**, NSE **(B),** CYFRA21-1 **(C)**, CA125 **(D)**, CA153 **(E)** and CA199 **(F)** in lung adenocarcinoma featured as solid nodules.

**Figure 5:**
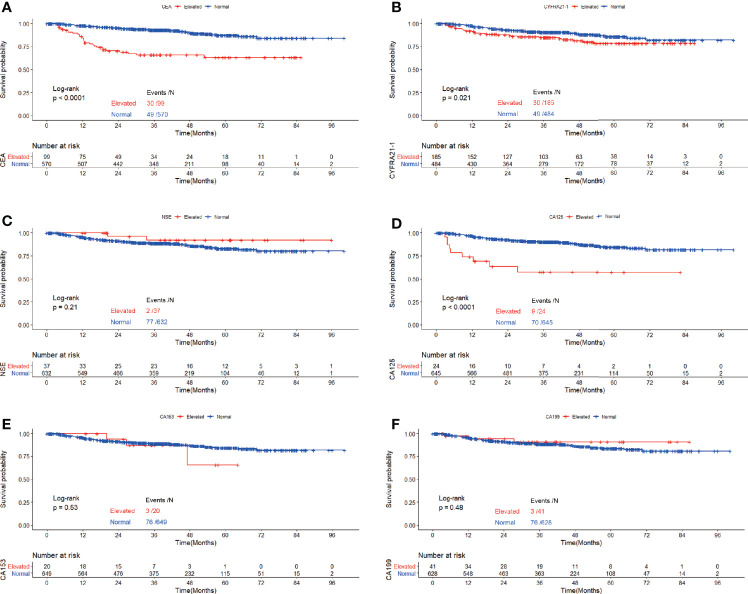
Recurrence free survival of patients with elevated *vs*. normal preoperative CEA **(A)**, NSE **(B),** CYFRA21-1 **(C)**, CA125 **(D)**, CA153 **(E)** and CA199 **(F)** in GGO-featured lung adenocarcinoma.

**Figure 4** depicted the association between serum tumor markers and RFS of patients diagnosed with adenocarcinoma presenting as solid nodules. Patients with elevated CEA, CYFRA21-1, NSE, CA125, CA153 and CA199 all showed worse RFS compared with those with normal serum tumor markers (CEA: P<0.0001, CYFRA21-1: P<0.0001, NSE: P=0.0027, CA125: P<0.0001, CA153: P<0.0001, CA199: P<0.0001) (**Figure 5**). Multivariable COX regression demonstrated that preoperative serum CYFRA21-1(HR=1.256, 95%CI:1.044-1.512, P=0.016) and CA125(HR=1.373, 95%CI: 1.050-1.795, P=0.020) were independent prognostic factors for patients with adenocarcinoma presenting as solid nodules while serum CEA was not (**Table 5**).

**Table 5:** Association between serum tumor markers and recurrence free survival of patients with radiologically solid adenocarcinoma.

Variables		Univariable		Multivariable	
		HR	P	HR	P
Sex	Male *vs*. Female	1.205 (1.012-1.435)	0.036		
Age		0.995 (0.987-1.004)	0.299		
Smoking History	Yes *vs*. Never	1.202 (1.004-1.438)	0.045		
Surgery	Lobar *vs*. Sublobar	1.624 (0.920-2.864)	0.094		
LVI	Present *vs*. Absent	2.272 (1.870-2.761)	<0.001	1.404 (1.143-1.726)	<0.001
VPI	Present *vs*. Absent	1.563 (1.305-1.872)	<0.001	1.351 (1.127-1.621)	<0.001
p-Size		1.238 (1.187-1.291)	<0.001	1.182 (1.122-1.246)	<0.001
Histology	HGADC *vs*. LGADC	1.217 (1.106-1.339)	<0.001		
T			<0.001		
	T1	1			
	T2	1.743 (1.448-2.099)	<0.001		
	T3	3.052 (2.240-4.159)	<0.001		
	T4	3.952 (1.948-8.018)	<0.001		
N			<0.001		<0.001
	N0	1		1	
	N1	2.064 (1.569-2.716)	<0.001	1.639 (1.238-2.170)	<0.001
	N2	4.100 (3.385-4.965)	<0.001	3.341 (2.725-4.097)	<0.001
CEA	Elevated *vs*. Normal	1.389 (1.273-1.516)	<0.001		
CYFRA21-1	Elevated *vs*. Normal	1.260 (1.154-1.375)	<0.001	1.256 (1.044-1.512)	0.016
NSE	Elevated *vs*. Normal	1.229 (1.073-1.408)	0.003		
CA125	Elevated *vs*. Normal	1.625 (1.439-1.835)	<0.001	1.373 (1.050-1.795)	0.020
CA153	Elevated *vs*. Normal	1.459 (1.285-1.658)	<0.001		
CA199	Elevated *vs*. Normal	1.273 (1.128-1.436)	<0.001		

HR, hazard ratio; CTR, consolidation to tumor ratio; LVI, lymph vascular invasion; VPI, visceral pleural invasion; HGADC, high grade adenocarcinoma; LGADC, low grade adenocarcinoma.

In the cohort of patients with adenocarcinoma presenting as GGO, log rank tests showed that elevated CEA(P<0.0001), CYFRA21-1(P=0.021) and CA125(P<0.0001) were associated with worse RFS while elevated serum NSE(P=0.21), CA153(P=0.53) and CA199(P=0.48) were not. Multivariable analysis revealed that elevated serum CEA (HR=2.160,95%CI:1.311-3.558, P=0.003) and CA125(HR=2.475,95%CI:1.163-5.266, P=0.019) both predicted worse RFS while elevated serum CYFRA21-1 did not (**Table 6**).

**Table 6:** Association between serum tumor markers and recurrence free survival of patients with GGO-featured adenocarcinoma.

Variables		Univariable		Multivariable	
		HR	P	HR	P
Sex	Male	2.245 (1.438-3.504)	<0.001	2.106 (1.339-3.311)	0.001
Age		1.004 (0.981-1.028)	0.712		
Smoking History	Yes	1.917 (1.217-3.022)	0.005		
CTR	CTR<0.5	1		1	
	0.5<=CTR<1	5.524 (2.540-12.013)	<0.001	3.390 (1.523-7.546)	0.003
Surgery	Lobar *vs*. Sublobar	1.459 (0.882-2.413)	0.141		
LVI	Present *vs*. Absent	6.115 (3.511-10.650)	<0.001	2.370 (1.272-4.415)	0.007
VPI	Present *vs*. Absent	1.756 (1.036-2.974)	0.036		
p-Size		1.494 (1.321-1.689)	<0.001	1.234 (1.055-1.464)	0.009
Histology	HGADC *vs*. LGADC	2.124 (1.350-3.343)	0.001		
T			<0.001		
	T1	1			
	T2	2.825 (1.742-4.579)	<0.001		
	T3	1.431 (0.198-10.348)	0.723		
	T4	14.924 (2.034-109.525)	0.008		
N			<0.001		0.002
	N0	1		1	
	N1	3.646 (1.455-9.133)	0.006	1.620 (0.619-4.242)	0.326
	N2	9.973 (5.740-17.328)	<0.001	3.075 (1.654-5.716)	<0.001
CEA	Elevated *vs*. Normal	2.125 (1.691-2.669)	<0.001	2.160 (1.311-3.558)	0.003
CYFRA21-1	Elevated *vs*. Normal	1.302 (1.038-1.635)	0.023		
NSE	Elevated *vs*. Normal	0.645 (0.320-1.302)	0.645		
CA125	Elevated *vs*. Normal	2.285 (1.614-3.235)	<0.001	2.475 (1.163-5.266)	0.019
CA153	Elevated *vs*. Normal	1.204 (0.676-2.145)	0.529		
CA199	Elevated *vs*. Normal	0.815 (0.458-1.451)	0.487		

HR, hazard ratio; CTR, consolidation to tumor ratio; LVI, lymph vascular invasion; VPI, visceral pleural invasion; HGADC, high grade adenocarcinoma; LGADC, low grade adenocarcinoma.

## Discussion

The prognostic significances of serum tumor markers in NSCLC still remained controversial. It was speculated that it might vary with radiological features and histological types. We reported the prognostic significances of six common-used serum tumor markers in surgically resected lung ADC and SCC respectively. We found that preoperative serum CEA, CYFRA21-1 and CA125 were independent prognostic factors for lung ADC while elevated preoperative serum CA199 was associated with poorer prognosis in lung SCC. Also, we reported the prognostic significances of preoperative serum tumor markers in lung ADC presenting as GGO for the first time. In lung ADC featured as GGO, elevated preoperative serum CEA and CA125 were associated with worse survival while elevated preoperative serum CYFRA21-1 and CA125 were associated with shorter RFS in lung adenocarcinoma presenting as solid nodules.

CEA, also known as carcinoembryonic antigen-related cell adhesion molecule-5 (CEACAM5), was a cell surface glycoprotein involved in cell adhesion. It always functioned as an adhesion molecule to promote metastasis of cancers. CEA was normally produced in human embryonic and fetal development (27). In normal adults, it was expressed in the colon, stomach, esophagus, appendix. Following malignant transformation, it was also detected in lung, ovarian, pancreas, gallbladder, colon and gastric cancers. In the serum of normal adults, CEA maintained a relatively low level (28).

A previous systemic review included 25 studies assessing the association between pre-treatment CEA level and survival of patients with NSCLC (29). Among the 19 studies in which the patients received surgical treatment, 15 studies reported that elevated preoperative serum CEA predicted worse survival for patients with surgically treated NSCLC while the other four studies did not. The conflicting results might be caused by the heterogeneity in histologic classification, disease stages and sample sizes of these studies. The authors found that an overweight of patients with SCC were observed in the studies with negative results. They implicated that preoperative serum CEA might play different prognostic roles in ADC and SCC. No study had demonstrated whether the prognostic value of serum CEA is comparable between ADC and SCC. Our study provided solid evidence that elevated preoperative serum CEA was associated with worse RFS in lung adenocarcinoma but not in lung squamous cell carcinoma.

CYFRA21-1, also referred to “keratin type I cytoskeletal 19”, was a kind of keratin intermediate filament proteins which were components of eukaryotic cytoskeleton. Serum CYFRA21-1 was measured to reflect the total tumor mass and the rate of cancer cell lysis as it was released from degraded cytoskeleton (30). Elevated CYFRA21-1 was reported to predict worse survival in patients with surgically treated NSCLC (5, 7, 8, 31–33). It was also reported to predict efficiency of treatment with EGFR-TKIs in advanced NSCLC (34). Nevertheless, the majority of the patients in these studies with positive results were diagnosed with ADC and the studies with a higher proportion of SCC reported conflicting results (35, 36). We implied that preoperative serum CYFRA21-1 was independent prognostic factor for patients with adenocarcinoma but not for patients with lung squamous cell carcinoma. Our study demonstrated that patients with an elevated CYFRA21-1 serum level had a shorter recurrence free survival than those with a normal serum level in patients with surgically treated lung adenocarcinoma. But the prognostic significance was not observed in patients with lung SCC. In our study, SCC showed a larger median tumor size than ADC which indicated that more necrosis might occur in SCC. It might lead to the high proportion of patients with an elevated level of serum CYFRA21-1 in SCC which might further result in its inefficiency in prognosis prediction. For radiologically solid ADC, elevated serum CYFRA21-1 might reflect tumor necrosis caused by aggressive growth which was associated with poorer survival.

NSE was a glycolytic enzyme mainly expressed in neuroendocrine tumors such as small cell lung cancer and large cell neuroendocrine carcinoma. In other histological classifications of NSCLC, elevated NSE might reflect its neuroendocrine differentiation or the presentation of SCLC components. In inoperable non-small cell lung cancer, some studies showed that a high serum NSE level was associated with a significantly worse prognosis (37–40). N Viñolas and colleagues found that a high pretreatment serum NSE level was associated with a more probability of response to treatment although it was also associated with a worse prognosis (41). Similarly, there were still some studies which came to a conflicting conclusion (42–44). A meta-analysis included 8 studies focusing on the prognosis value of NSE in NSCLC was published in 2014. The patients in all the 8 studies were treated with chemotherapy with or without radiotherapy combined. It indicated that serum NSE level was not a significant prognostic factor for NSCLC (45). In surgically treated NSCLC, controversial still remained regarding the prognostic value of serum NSE. Dangfan Yu and colleagues found that a high preoperative serum NSE portended a worse survival in operable NSCLC (6). Shouying Li and colleagues demonstrated that a high level of NSE was associated with a worse survival in surgically resected lung adenocarcinoma patients harboring anaplastic lymphoma kinase rearrangements (46). However, the majority of previous studies found that the prognostic value of preoperative serum NSE in surgically treated NSCLC was limited (5, 8, 47–49). Our study indicated that elevated preoperative serum NSE did not indicate a worse survival in completed resected lung adenocarcinoma an SCC which was consistent with the majority of previous studies.

CA199, also known as sialyl Lewisa antigen, was mainly utilized in the diagnosis and treatment of pancreatic carcinoma nowadays (50, 51). It was deduced to involve in the extravasation of cancer cells from blood to distant organs. CA199 was expressed in normal human epithelial tissues of pancreas, gall bladder, stomach, bronchus, ovary and fallopian tube. In the blood of cancer patients, the elevated CA199 level reflected its production by and release from cancer cells (52). Toshiaki Kawai et al. found that serum CA199 was correlated with survival in advanced lung adenocarcinoma but not in early-stage lung adenocarcinoma (53). J Niklinski and colleagues found that elevated serum CA199 indicated worse survival in patients with NSCLC (54). Due to the scarcity of studies focused on the prognostic significance of serum CA199 in NSCLC, the prognostic significance of serum CA199 in NSCLC was always neglected. In our study, preoperative serum CA199 was not corrected with RFS in patients with lung adenocarcinoma in the multivariable analysis while it was an independent prognostic factor for patients with lung SCC. The prognostic value of serum CA199 in lung SCC was a new finding, we were also cautious about this result. External validations might be required to confirm this result. Also, further studies were required to reveal the mechanism underneath.

Our study showed that elevated preoperative serum CA125 was associated with poorer RFS in lung adenocarcinoma regardless of its radiological appearance. CA125 was mainly utilized in the management of ovarian carcinoma (55). It was a peptide epitope of a membrane-spanning mucin MUC16 which promoted cancer cell proliferation and inhibited anti-cancer immune responses. MUC16 was expressed in normal ovarian, endometrial, corneal and bronchial epithelial cells. It was overexpressed in multiple malignant tumor types including breast, pancreatic, colorectal and lung cancer. MUC16 was released from the cell surface following proteolytic cleavage and rapidly processed by the reticulo-endothelial cells in circulation, leaving behind debris of the mucin in circulation to be detected such as the CA125 (56). The studies of Pollan and Yu suggested that elevated preoperative serum CA125 was related to poorer outcome in patients with NSCLC (6, 57). Ma and colleagues analyzed 164 patients with surgically resected NSCLC of stage I and found that no significant difference of survival was observed between patients with an elevated preoperative serum CA125 level and those with a normal level (8). The prognostic value of CA125 was still in controversial with these conflicting results. Our study provided solid evidence that elevated preoperative serum CA125 was associated with poorer survival both in GGO-featured adenocarcinoma and radiologically solid adenocarcinoma.

With immunotherapy widely used in advanced lung cancer, a series of studies had focused on the biomarkers such as PD-L1 expression and tumor mutation burden (TMB) to predict the efficiency of immune checkpoint inhibitors (58–63). These immunological parameters were also used in prognosis prediction in patients with surgically resected NSCLC. Siddhartha Devarakonda and colleagues reported that high TMB was associated with a better prognosis (64) while Yuki Owada-Ozak and colleagues reported conflicting results (65). A large cohort study from South Korea revealed that PD-L1 expression might be associated with poor prognosis in patients with resected NSCLC though the significance weakened when postoperative treatment was taken into consideration (66). The association of serum tumor markers and these immunological characteristics was unclear. Further study was recommended to include these immunological parameters to reduce the bias caused by imbalance in immunological characteristics. The interaction between serum tumor markers and tumor immune microenvironment might also require further study to explore.

With the finding that preoperative serum tumor markers could provide more information regarding the probability of recurrence, assay of preoperative serum tumor markers such as CEA, CYFRA21-1, CA125 and CA199 might be recommended in routine clinical practice in the future. For patients with lung ADC, more intensive follow-up strategy should be utilized for patients with elevated preoperative serum CEA, CYFRA21-1 or CA125. Similarly, more careful follow-up strategy should be also utilized in lung SCC patients with elevated serum CA199. On the other hand, for patients of stage IB, the adjuvant chemotherapy might be guided by the level of preoperative serum tumor markers in some ways. For patients diagnosed with GGO, sublobar resection might be precluded by an elevated preoperative serum CEA or CA125. Surgeons should take preoperative serum CEA and CA125 into account when planning the operation procedure for GGO. Further studies were required to validate these perspectives.

Some limitations of this study must be noticed. It was a single institutional study and the selection bias seemed to be inevitable. The selection bias might also be introduced by the exclusion of AIS, MIA and LPA in the analyses. Also, the data were collected retrospectively and the recall bias might have an influence on the final results. The median follow-up time was only 41 months which might lead to inadequate events to perform overall survival analyses. Finally, data of immunological characteristics were missing in this study, hence potential imbalance in immunological characteristics might also affect the results.

## Conclusions

In conclusion, elevated preoperative serum CEA, CYFRA21-1 and CA125 were associated with worse RFS in patients with surgically resected lung adenocarcinoma while CA199 was in patients with surgically resected lung SCC. In patients with radiologically solid adenocarcinoma, elevated preoperative serum CYFRA21-1 and CA125 predicted worse RFS and elevated preoperative serum CEA and CA125 were independent prognostic factors for patients with GGO-featured adenocarcinoma. The prognostic significances of preoperative serum tumor markers in non-small cell lung cancer varied with radiological features and histological types.

## Data Availability Statement

The raw data supporting the conclusions of this article will be made available by the authors, without undue reservation.

## Author Contributions

HC: Conceptualization, data curation, formal analysis, writing - original draft, and writing - review and editing. FF: Data curation, methodology, and writing - review and editing. YZ: Data curation, project administration, and writing - review and editing. HW: Data curation, Writing - review and editing. HH: Project administration, supervision, and writing - review and editing. YS: Supervision, and writing - review and editing. YawZ: Supervision, and writing - review and editing. XJ: Supervision, and writing - review and editing. YanZ: Conceptualization, methodology, project administration, writing - original draft, investigation, and writing - review and editing. All authors contributed to the article and approved the submitted version.

## Funding

This work was supported by the National Natural Science Foundation of China (81930073), Shanghai Municipal Science and Technology Major Project (Grant No. 2017SHZDZX01, VBH1323001/026), Shanghai Municipal Key Clinical Specialty Project (SHSLCZDZK02104), and Pilot Project of Fudan University (IDF159034).

## Conflict of Interest

The authors declare that the research was conducted in the absence of any commercial or financial relationships that could be construed as a potential conflict of interest.
